# A narrative review of the synthesis, characterization, and applications of iron oxide nanoparticles

**DOI:** 10.1186/s11671-023-03898-2

**Published:** 2023-10-10

**Authors:** Joseph Ekhebume Ogbezode, Ucheckukwu Stella Ezealigo, Abdulhakeem Bello, Vitalis Chioh Anye, Azikiwe Peter Onwualu

**Affiliations:** 1 Department of Materials Science and Engineering, African University of Science and Technology, Abuja, Nigeria; 2 Department of Mechanical Engineering, Edo State University Uzairue, Uzairue, Edo State Nigeria; 3 Centre for Cyber-Physical Food, Energy and Water System (CCP-FEWS), Electrical and Electronic Engineering Science, University of Johannesburg, Johannesburg, South Africa; 4 Department of Theoretical and Applied Physics, African University of Science and Technology, Abuja, Nigeria

**Keywords:** Green synthesis, Iron oxide nanoparticles, Characterization, Applications

## Abstract

The significance of green synthesized nanomaterials with a uniform shape, reduced sizes, superior mechanical capabilities, phase microstructure, magnetic behavior, and superior performance cannot be overemphasized. Iron oxide nanoparticles (IONPs) are found within the size range of 1–100 nm in nanomaterials and have a diverse range of applications in fields such as biomedicine, wastewater purification, and environmental remediation. Nevertheless, the understanding of their fundamental material composition, chemical reactions, toxicological properties, and research methodologies is constrained and extensively elucidated during their practical implementation. The importance of producing IONPs using advanced nanofabrication techniques that exhibit strong potential for disease therapy, microbial pathogen control, and elimination of cancer cells is underscored by the adoption of the green synthesis approach. These IONPs can serve as viable alternatives for soil remediation and the elimination of environmental contaminants. Therefore, this paper presents a comprehensive analysis of the research conducted on different types of IONPs and IONP composite-based materials. It examines the synthesis methods and characterization techniques employed in these studies and also addresses the obstacles encountered in prior investigations with comparable objectives. A green engineering strategy was proposed for the synthesis, characterization, and application of IONPs and their composites with reduced environmental impact. Additionally, the influence of their phase structure, magnetic properties, biocompatibility, toxicity, milling time, nanoparticle size, and shape was also discussed. The study proposes the use of biological and physicochemical methods as a more viable alternative nanofabrication strategy that can mitigate the limitations imposed by the conventional methods of IONP synthesis.

## Introduction

### Study background

The production of atomic and molecular particles between 1 and 100 nm is known as nanotechnology and nanoscience, respectively [1, 2]. Nanomaterials are substances that exhibit the characteristics of microscopic objects. Optics [3, 4], mechanics [5, 6], bioengineering [6, 7], medicine [8, 9], and environmental remediation [10, 11] are all engineering disciplines that utilize these particles. According to the literature [9–11], numerous strategies have been employed to produce metallic nanoparticles (MNPs). Nanoparticles (NPs) are often produced using chemical [12], biological [13], and mechanical [14] techniques. Physical and chemical processes are used to produce metal/metal oxide nanoparticles [15]. However, the limitations of these techniques reveal that the synthesis of metal and metal oxide nanoparticles necessitates the use of toxic, environmentally unfriendly, and highly reactive reducing agents [16]. This biological compound includes sodium hydroxide, hydrazine hydrate, wheat, glucose, xylose, and vitamin C [17]. Given that nanoparticle synthesis relies on the use of nontoxic methods to avoid the involvement of hazardous chemical agents [18], it is imperative for researchers to consistently endeavor to develop highly efficient biochemical reduction agents and acquire environmentally friendly resources for the production of nanoparticles.

Extensive research has been conducted on iron nanoparticles (FeNPs) [19, 20] due to their promising application in various fields, including medicine, environmental remediation, and sewage treatment. In addition, the process of water purification can be effectively achieved by using nanoscale and nanofiltration techniques [21, 22]. Recent advancements in nanotechnology have examined the importance of removing toxic compounds from nanoparticles by using an environmentally friendly technique. For instance, iron nanoparticles (FeNPs) in water and the environment compete favorably with hazardous chemical contaminants [23]. Utilizing environmentally friendly nanoparticle production processes has the potential to expedite advancements in the biological, economic, and technical domains [24]. This article provides an assessment of the economic, biological, and environmental implications linked to the production of iron and iron oxide nanoparticles, considering their extensive range of potential applications.

### Synthesis of iron oxide nanoparticles

Considering the wide range of physical and chemical properties exhibited by IONPs, researchers continue to show substantial interest in their production and application. This is due to the various structural and non-structural uses that IONPs provide [25]. For instance, IONPs have various properties, including strong magnetic behavior, phase microstructure, mechanical and thermal properties, etc. These properties indirectly influence their suitability for use in scientific and engineering fields as well as multidisciplinary purposes. Many works of literature have attempted to identify and delineate the different systematic approaches used in the synthesis and applications of IONPs. To appreciate the usage of nano-materials, there is a need to conduct an in-depth review of existing research on metal oxide nanoparticles and nanocomposite-related articles. However, the state of the art in nanoparticle research encompasses their diverse applications, while the selection of nanoparticles is driven by their exceptional properties. This work's novelty stems from its innovative synthesis approach, addressing a knowledge gap in precise nanoparticle control, and its contribution to advancing energy storage applications. The knowledge gap further expositions of recent trends in nano-synthesis, and the application of IONPs in modern-day nanotechnology has been enumerated in the study. Despite the notable strides in nanoparticle research, several knowledge gaps persist. A prominent gap lies in the synthesis of nanoparticles with precise control over their characteristics, particularly regarding size, shape, and surface chemistry. Furthermore, understanding the long-term effects of nanoparticles on human health and the environment remains a challenge, prompting the need for safer and more sustainable synthesis methods. Therefore, this paper presents a comprehensive overview of recent articles that have made significant contributions to the advancement of knowledge in the areas of synthesis, characterization, nanofabrication, and applications of IONPs.

#### Overview of research articles on IONPs and their significance

The significance of synthesized IONPs cannot be overemphasized due to their extensive potential applications in various disciplines such as science, engineering, and medicine. The application of IONPs includes but is not limited to electronic appliances, automobiles, biomedicine, water purification, environmental remediation, and drug delivery systems. Saif et al. [26] conducted a green synthesis of zero-valent metallic iron (ZVMI) from hematite and magnetite and evaluated the ecotoxicological contrast between green and non-green iron nanoparticle synthesis. The study provides a detailed summary of the latest advancements in nanoscience and nanotechnology in the production of new nanomaterials (NMs). The purpose of the study explains the negative impact of supplementary and derivative chemicals on nanofabricated materials. The review validates the green synthesis strategy for the production of zero-valent metallic iron (ZVMI) from hematite and magnetite, as well as their application in environmental pollution and the water filtration process. The paper also provided an ecotoxicological contrast between green and non-green IONP synthesis.

Natarajan et al. [27] describe numerous approaches for producing magnetic iron oxide nanoparticles (MIONPs) for biomedical applications, surface modification, and cytotoxic effects. It also provides a comprehensive understanding of the potential state-of-the-art technique required to mitigate the cytotoxic effect of MIONP free radicals in industrial and biomedical applications. In addition, it provides a viewpoint on surface-modified MIONPs in medicinal and industrial applications for general well-being.

Rosen et al. [28] describe the use of functionalized superparamagnetic iron oxide nanoparticles (SPIONs) for cancer and solid tumor diagnosis and treatment. The study concluded that functionalized SPIONs techniques are superior to magnetic resonance imaging for cancer and solid tumor diagnosis and treatment.

Kolhatkar et al*.* [29] highlight the unique production and application of different nanoparticles. The shapes, sizes, and elemental composition of these magnetic nanoparticles were studied. This research also provided insight into several methods of adjusting magnetic nanoparticles for greater effectiveness and specific use. Khan et al*.* [30] provided a comprehensive review of the synthesis, application, and toxicity of nanoparticles made from various materials (ceramics, metals, polymers, etc.) with particle sizes between 1 and 100 nm. The characteristics of produced nanoparticles are dependent on their size, shape, and crystalline structure. The presented knowledge supports the potential of the produced nanoparticles for extensive use in medical, imaging, energy-based research, and environmental applications.

Jalil et al. [31] synthesized hematite (Fe_3_O_4_) NPs from its natural iron ore using a mechanical alloying process. The morphological, mineralogical, and phase composition of the produced Fe_2_O_3_ samples were analyzed by SEM, XRD, and XRF. The results of XRD and XRF analyses of the synthesized sample exhibit similar and consistent properties. The study demonstrates that the nano-hematite (Fe_2_O_3_) phase produced by mechanical or ball milling is crucial to the magnetic behavior of natural iron ore sources for the production of electromagnetic devices.

Ganapathe et al*.* [32] synthesized magnetite (Fe_3_O_4_) NPs using a ball-milling process. The results of the XRD and XRF analyses exhibit similar and consistent properties. Concise information about the most recent methodologies and developments in magnetite nanoparticle synthesis, with a focus on their possible applications. The article provided a platform for future research by expanding our understanding of the possible applications of new organic and inorganic materials through case studies of their use in vitro and in vivo. Ali et al*.* [33] provided an overview of recent advancements in the synthesis, characterization, and applications of iron oxide nanoparticles with enhanced characteristics. The paper provides a comprehensive summary of IONP preparation processes, their applications, and potential limitations. The objective of this study was to provide additional extensive data on the present research interest in IONP production, from synthesis to characterization and application. Petrovský et al*.* [34] investigate the magnetic properties of magnetite nanoparticles produced from the stoichiometric combination of hematite ore samples with metallic iron particles in an inert atmosphere using the mechanical ball-milling technique for a predetermined time. It also provides additional support for the influence of magnetic properties and hardness on magnetite NPs produced by ball milling in the presence of a hot inert gas atmosphere. This is based on their relevance and applications in water filtration and environmental remediation. Thus, the metal-ion-sensing potential of IONPs made it possible for their usage in heavy metal elimination from polluted water due to their minor size, magnetic potential, and larger surface area.

Singh et al. [35] synthesized nanocrystalline FeCr alloy using the mechanical alloying method. The review is based on the need to probe the challenges involved in the thermal processing of synthesized iron-alloyed nanoparticles using oxidation-resistant nanocrystalline materials. It was concluded that it was necessary to troubleshoot the structural defects in nanocrystalline FeCr alloy. It is quite imperative to introduce similar nanostructure alloys to ascertain grain compactness within the metal matrix structure of the base particle. Mohapatra et al*.* [36] provide a comprehensive review of the preparation and application of α-Fe_2_O_3_ NPs synthesized by precipitation method. Iron oxide properties such as goethite (α-FeOOH), hematite (α-Fe_2_O_3_), and maghemite (γ-Fe_3_O_4_) were synthesized according to their size, crystalline structure, and magnetic properties. The synthesized iron oxide particles were extensively substituted in biomedical, catalytic materials, magnetic recording devices, wastewater treatment, and medicine. The review explains the basic classifications of nanoparticles, the top-down approach and bottom-up approach, and the different forms of iron nanoparticle synthesis. It also explains how iron oxide nanoparticles can be synthesized with less energy utilization and decreased particle size.

Irfan et al*.* [37] explain the basic classifications of NPs (i.e., organic, ceramic, non-organic, and carbon-based NPs) and the kind of technological basis for their synthesis and characterization. The top-down approach and the bottom-up approach are the two major techniques used in nanoparticle synthesis. Also, metal and metal oxide nanoparticles (MONPs) are direct sub-classifications of inorganic nanoparticles. Classification of nanoparticles into one-dimension, two-dimension, and three-dimension forms is done. The basic techniques and novel applications of different nanoparticles based on their mechanical properties were reviewed.

Guo et al*.* [38] reviewed the basic techniques and novel applications of different nanoparticles based on their mechanical properties. Special nanoparticle mechanical properties vis-à-vis their interracial forces acting on the surface and the basics of physics were explained. Young modulus, hardness, friction, and abrasion were surveyed, as well as the parameters involved in reinforced or coated nanoparticles and nanofabrication operations. The study gave a summary and future perspective on the effect of mechanical properties, and the parameters were involved in reinforced or coated NPs and nanofabrication operations.

Satyanarayana, et al*.* [39] also reviewed various techniques for the preparation of nanomaterials, which were enumerated. Synthesized NPs, which include mechanical, electrochemical, sonochemical, micro-emulsion, and physical and chemical reduction, were discussed in detail. The paper postulates a future perspective into research for NP synthesis, operations, production strategy, and their applications.

Ali et al*.* [40] further gave a comprehensive review of the synthesis, applications, and challenges involved in the production of iron oxide nanoparticles. The review further enumerates the various methods of iron oxide nanoparticle synthesis according to works of literature, with emphasis on biocompatibility, magnetic characteristics, and morphology control. The report revealed that magnetic iron oxide nanoparticles (MIONPs) with good magnetic susceptibility are better applied in biomedical research, drug delivery, and pharmaceutics. The study was designed to give up-to-date information on the synthesis, characterization, and applications of iron nanoparticles. Samrot et al*.* [41] reviewed the numerous fields of applications of SPION that were elucidated. The various methods of SPION synthesis, which include physical, chemical, and biological methods, were also elaborated. The report shows that the synthesis and characterization of SPIONs are properties- and feature-based. The exceptional magnetic property of SPIONs encourages its application as a contrast agent in magnetic resonance imaging and for biomedical purposes. The report also elaborated on the synthesis and application of surface-functionalized MIONPs. This nanomaterial has elaborate applications in biomedical, drug delivery, environmental remediation, radiation therapy, tissue engineering, etc. Wu et al*.* [42] elaborated on the synthesis and application of surface-functionalized magnetic iron oxide nanoparticles (SFMION), which have wide applications in the field of biotechnology. Recent developments in the preparation strategies and practical applications of SFMION were elaborately discussed. The properties of functionalized iron oxide nanoparticles, such as stability, biocompatibility, and surface function characteristics, were also briefly discussed. The study was concluded by enumerating the major challenges associated with the production and synthesis of the NPs, while future perspectives for research were suggested.

Dobson [43] reviewed the use of synthesized IONPs for magnetic devices and drug delivery systems and stressed the need for further studies in gene and drug delivery operations. The study provided an explanation of the use of various magnetic NP designs for biomedical purposes, including clinical and animal trials. The contribution to nanoparticle research established the need for further studies in gene and drug delivery operations. The report stressed the need to further advance the applications of magnetic nanoparticles for tumor treatments and clinical applications. Wu et al*.* [44] review focuses on the strategies and development of recent advances in the synthesis, characterization, and applications of functionalized IONPs in biomedicine. The study considers the various challenges, functionalized strategies, and problems involved in the bio-applications of IONPs and their future research prospects. Xu et al*.* [45] explained the recent advances in the use of iron oxide nanoparticles for wastewater treatment and gave future research perspectives on the likely challenges and solutions. Iron oxide coated with gold nanoparticles was synthesized using the sonochemistry method. The paper explains the recent advances in the use of IONPs as a cleanup technological strategy for wastewater treatment in the modern age. The study bridges the gaps and limitations involved in the various synthesis, characterization, and application of IONPs for water purification. The work also gave future research perspectives on the likely challenges and solutions that may affect the continued use of such water purification methods and their environmental impact.

**Fig. 1:**
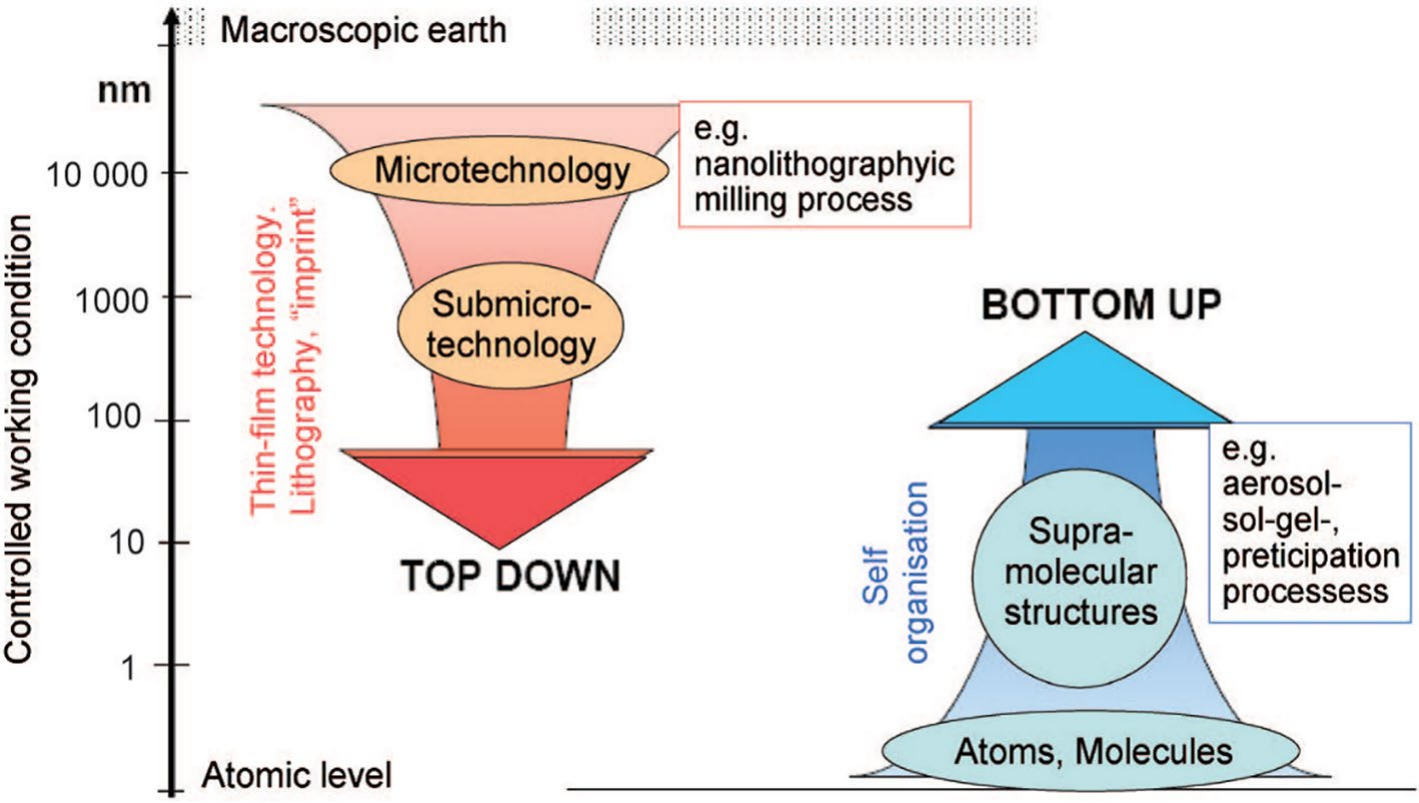
Top-down and bottom-up methods of nanoparticle production [25] Copyright 2011: NanoTrust-Dossier

Gul, et al*.* [46] attempted to substantiate the recent advances in the production and application of magnetic IONPs as a theranostic agent. Several methods of synthesizing magnetic iron oxide nanoparticles were enumerated, and the connections between the synthetic routes, magnetic properties, and size of the nanoparticle were extensively investigated. Generally, the importance of IONPs and composites is enshrined in their vast application in wastewater treatment, environmental remediation, biomedical and electrical appliances, microchips, superparamagnetism, magnetic resonance imaging (MRI), cancer treatment, etc. The extent of their importance is based on various factors, which vary from IONP type, size, shape, magnetic properties, physical properties, morphological tendencies, etc. For instance, the synthesis of inorganic metals coated with IONPs has gained research attention in recent times with applications in cancer cell eradication and biomedical processes. Dheyab, et al. [47] performed a comprehensive review of the synthesis of iron oxide coated with gold nanoparticles (Fe_3_O_4_@AuNPs) using the sonochemistry method. This involves the modification of chemical reactions using ultrasound. The study addresses the fundamental principles of the sonochemistry approach. It also described the properties of the synthesized IONP products using the sonochemistry method through the summary of key works of literature from relevant publications using case studies such as Fe_3_O_4_NPs, AuNPs, and Fe_3_O_4_@AuNPs [48–50]. A simple, rapid stabilization process of Fe_3_O_4_ NPs using the citric acid modification method was conducted by Dheyab et al. [51]. The study employed the rapid method of one-step co-precipitation to synthesize magnetite NPs in the presence of magnetized citric acid in the form of an aqueous colloidal solution (Fe_3_O_4_@CA). The as-prepared Fe_3_O_4_@CA had a Zeta potential value increment from − 31 to − 45 mV due to its high magnetic saturation capacity. Thus, the synthesized IONPs do possess a high magnetic saturation value of 54.8 emu/g, which in turn leads to promising sustainability for biomedical applications. These IONPs can be used as theranostic agents for cancer cell eradication, biomedical processes, and drug delivery systems. Several studies also attempted to use the sonochemical approach for the synthesis of Fe_3_O_4_@Au NPs used for cancer cell eradication via magnetic resonance imaging (MRI) and computed tomography. The IONPs synthesized in such gold (Au) powder were precipitated to produce NPs with a size of 22 nm in 5 min and have proven to be efficient as a theranostic agent through a sonochemical approach for cancer cell eradication [52, 53]. The study concluded because of the potential applicability and future research prospects of IONPs and their importance as theranostic agents for cancer cell eradication, biomedical processes, and drug delivery systems. For easy understanding, this paper specified that the production of IONPs was classified into two different ways based on their method of preparation and application in various fields of study, as depicted in Fig. 1.

**Fig. 2:**
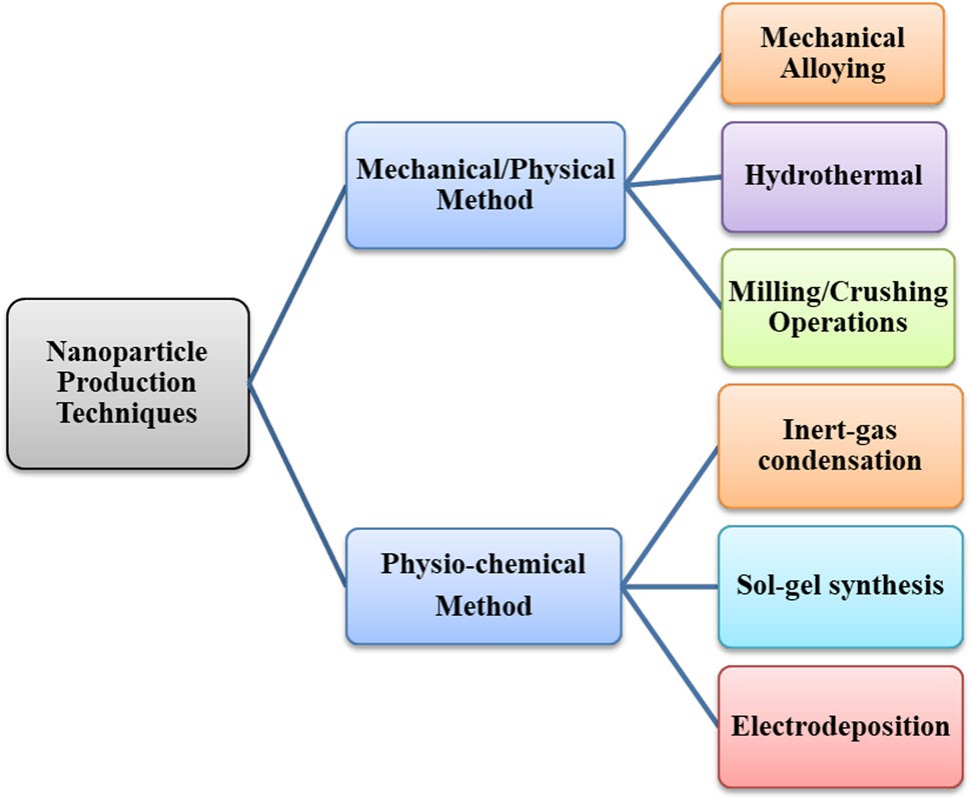
Overview of the production processing of nanomaterials

The two basic strat**e**gies for the production of nanomaterials are top-bottom and bottom-up approaches. The top-bottom method involves mechanical crushing of the base materials, while the bottom-up method involves a chemical process to alter the structure of the base materials, depending on the chemical characteristics of the nanoparticles as depicted in Fig. 1. Also, Fig. 2 shows a comprehensive summary on the production route for metal/metal oxide nanoparticles from the known conventional methods.

### The top-bottom/mechanical production method

For the generation of NPs, the top-down/mechanical production method employs a mechanical/milling crushing process that is time-consuming and energy-intensive [54, 55]. This procedure is time-consuming and employs mechanical abrasion procedures that are energy-intensive. Metallic oxide continues to be the conventional source of material that is pulverized by a stoichiometric operation before being crushed using high-energy ball mills [56]. The mills are manufactured from a high-strength steel material with superior mechanical hardness [57]. As depicted in Fig. 2, the mechanical/physical/crushing method produces nanoparticles that are considerably larger than those produced by the chemo-physical method.

### The bottom-up/chemical production method

The bottom-up/chemical production method involves co-precipitation and aerosol activities and generates nanomaterials with superior geometric structures and size ranges. These nanomaterials are formed by homogeneous nucleation via the solid–liquid reaction or by condensation reaction in the gas–solid reaction.

**Fig. 3:**
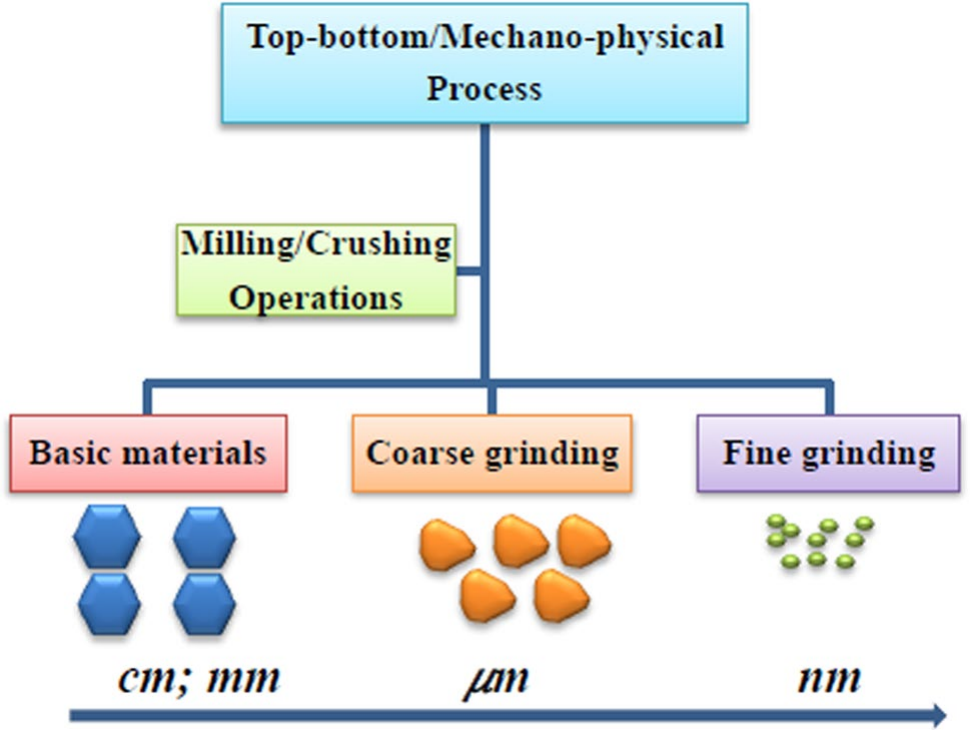
Overview of nanomaterial production by mechanophysical process

**Fig. 4:**
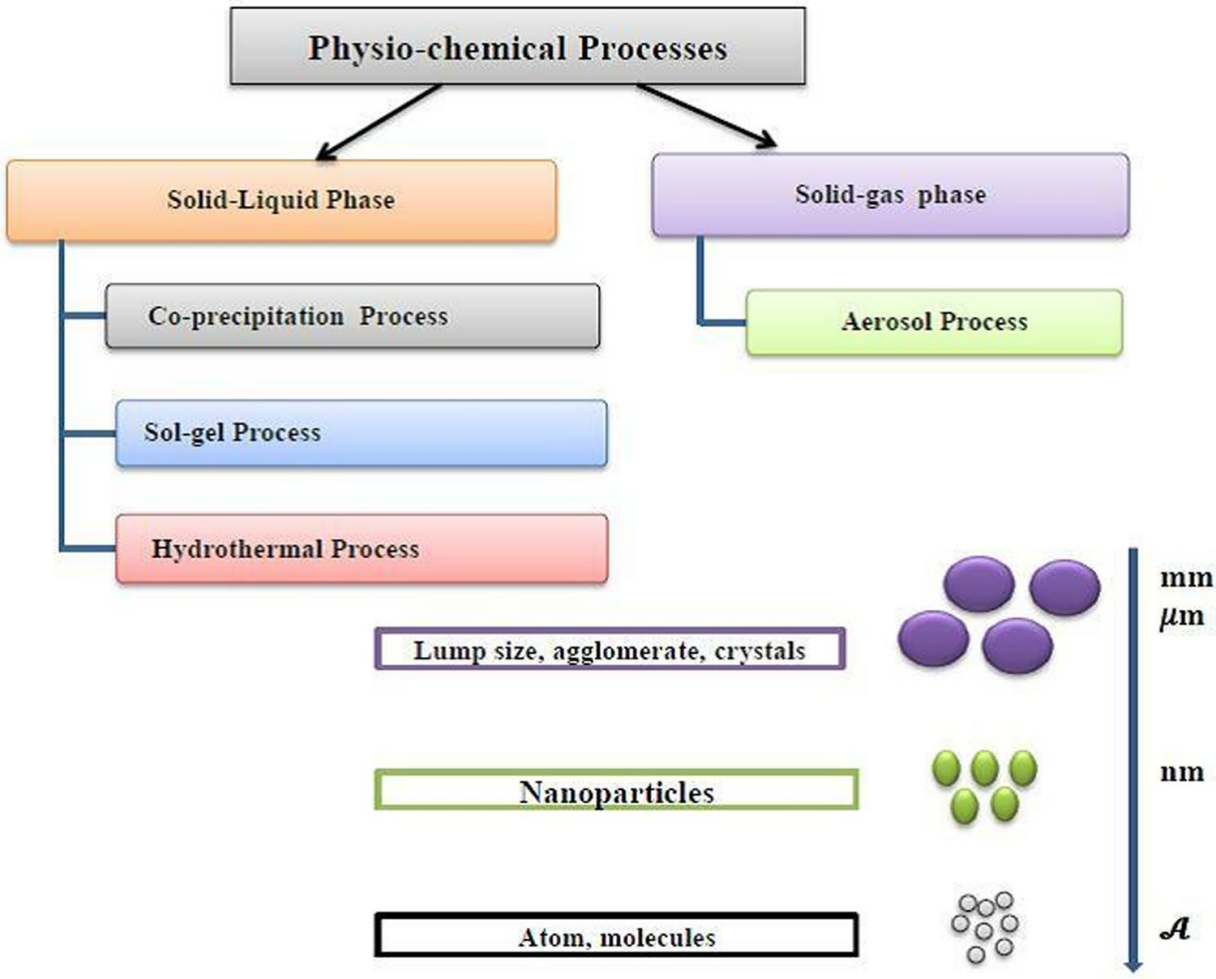
Overview of nanomaterial production by physiochemical process

This method also involves an atomic-molecular interaction between a nanoparticle of base metallic oxide and a chemical reducing agent. This technique generates a sophisticated strategy for the synthesis of nanoparticles, which takes delight in its ability to generate nanomaterials with superior geometric structure and size ranges [58]. Figure 3 depicts the schematics of IONPs synthesis using co-precipitation and aerosol activities. Nanoparticles produced using any of the aforementioned physiochemical processes are formed either by homogeneous nucleation via the solid–liquid reaction or by condensation reaction in the gas–solid reaction, as well as particle coalescence technique from particle fusion, which has applications in wall reactors, plasma jets, and carbon nanotubes [59]. Through physiochemistry, as shown in Fig. 4, nanoparticles created by gas–solid chemical reactions yield vapor product materials [60].

## Conventional routes for IONPs synthesis and extraction

Over the decades, different authors have postulated various methods for the synthesis and characterization of IONPs [61–65], including physical [66], chemical [67], or biological [68]. Recently, a few pieces of literature have attempted to combine two or more methods of the known conventional technique of synthesizing nanoparticles [69–72]. Physicochemical, biochemical, and biophysical approaches are included. No published effort has been able to simultaneously synthesize and characterize IONPs utilizing all three conventional approaches. This is indeed a new area of nanotechnology research in which researchers can advance the applications of nanoparticles beyond the known fields of study, as the literature demonstrates that the method employed in the synthesis of nanomaterials determines their quality in terms of sizes, shapes, magnetic behavior, surface functionality, and applicability [73, 74]. Listed below are several conventional techniques for producing nanomaterials. For the sake of clarity, a systematic review of the synthesis and characterization of iron oxide nanoparticles (IONPs) was performed. The study also gives a holistic background on the various production routes of IONPs and investigates their nanocrystalline phase structure and characteristics in real-time experiments. It also summarizes diverse applications of IONPs and iron oxide nanocomposite-based materials in wastewater filtration, environmental remediation, plant development, drug delivery, bone repair, cancer cell eradication, tissue regeneration, and the biomedical field. Additionally, the influence of their mineralogical properties, morphology, phase structure, biocompatibility, magnetic properties, toxicity, nano-processing time, nanocrystalline size, and shape was also discussed.

## Synthesized iron oxide nanoparticles’ mechanical alloying method

This method for extracting NPs of iron oxide is an energy-intensive procedure that includes a grinding medium, making it a completely mechanical operation [75]. A ball-mining machine is used to crush natural iron oxide particles into polycrystalline NPs less than 5 nm in size [76]. The milling procedure exerts abrasive force for approximately 23 h. The approach is capable of providing a nanoscale particle size reduction impact on raw iron oxide material [77]. The ball-milling machine utilized for the mechanical alloying of iron oxide particles is rotated at 300 rpm for 20 h, and the morphological and microstructural changes of the grounded iron oxide particles are examined using XRD and high-resolution SEM micrographs [78]. Using X-ray fluorescence (XRF) and energy-dispersive spectroscopy (EDS), the elemental composition of the ground FeNPs was examined. Using a magnetic detecting device, such as the vibrating-sample magnetometer, reveals other properties, such as magnetic behavior (VSM).

### Synthesized iron oxide nanoparticles by mechanochemical method

A solid-state chemical reaction technique is used to synthesize iron oxide nanoparticles by collective annealing and milling. Iron oxide particles are annealed at temperatures between 300 and 700 degrees Celsius [79]. Using a ball-milling machine, the iron oxide particles are crushed into powered NPs with an average size range of 10 to 100 nm. The produced NPs can be evaluated utilizing techniques such as X-ray diffractometry (XRD), X-ray fluorescence (XRF), scanning electron microscopy (SEM), electro-dispersive spectrometry (EDS), thermogravimetric analysis (TGA), and differential thermal analysis (DTA). In recent years, the mechanochemical approach has been used to create iron oxide nanoparticles due to their unusual chemical and physical properties [80]. This method is easy, has a high yield scale, and has minimal economic consequences, but could present a difficulty in their post-processing stages and applications [81]. Mechanochemical nanoparticle processing of iron/iron oxide is a novel technology for iron ore production and characterization. The method combines concurrent procedures that combine ball-milling and solid-state displacement methodologies with a low energy demand and a high propensity toward environmental friendliness [82].

However, the development of the mechanochemical approach to NPs for large-scale manufacturing could present a difficulty in their post-processing stages and applications [83]. Several industrial preparations of iron oxide nanoparticles have been done to improve the nanomaterials' physical and magnetic properties [84, 85]. This method is restricted to the anticipated industrial uses of the synthesized iron nanoparticles, such as pigmentation [86], catalysis [87], wastewater treatment [88], gas purification [89], combustion [90], electrochemical [91], co-precipitation [92], and superparamagnetism [93]. In light of this, the mechanochemical approach remains a pollution-free and eco-friendly physical technique due to its absence of organic solvents and ecological cleanliness [94]. Consequently, the primary objective of this iron nanoparticle manufacturing method is to manufacture nanoparticles that are environmentally friendly and support the green synthetic approach to nanomaterial fabrication.

### Synthesized IONPs by chemical and biological methods

The importance of producing green nanomaterials with little environmental impact through the use of green technology cannot be overstated. There is a need to commence laboratory-scale nanoscience-related research due to the constraints of massive material acquisitions and their cost implications, which are key obstacles to the sustainability of nanodevice manufacture in the engineering industry. The chemical and biological methods of nanoparticle synthesis are commonly employed in nanosciences to synthesize, extract, and manufacture nanomaterials for laboratory-scale research experiments [95]. This technology has demonstrated its potential for generating environmentally friendly nanomaterials with a minimal economic impact on end-users [96]. Over the years, several biochemical methods have been used to generate nanoparticles from bulk materials, including co-precipitation, hydrothermal, sol–gel, inert gas condensation, microwave-assisted method, micro-emulsion, sputtering, chemical vapor deposition, sonochemical vapor deposition, physical vapor deposition, liquid infiltration, rapid solidification based on their atomic and molecular structure [97]. This is made feasible by introducing additional particles into the metal matrix of the base materials with the aid of biological and chemical reagents [98]. At specific chemical energies, different ionic exchanges occur to restructure and rearrange the microstructure characteristics of the basic materials [99–102]. Numerous published publications have utilized the various biochemical methods of nanoparticle extraction to manufacture even more challenging engineered materials [103–106]. In this study, the biochemical approaches for manufacturing NPs of iron and iron oxide were examined. According to Iqbal et al. [107] and Issa et al. [108], the biochemical techniques used to synthesize iron oxide nanoparticles include co-precipitation, hydrothermal, sol–gel, inert gas condensation, microwave-assisted method, micro-emulsion, sputtering, chemical vapor deposition, sonochemical vapor deposition, physical vapor deposition, liquid infiltration, rapid solidification, and so on. Baaziz et al. [109] generated iron oxide nanoparticles in coated sand using the biochemical approach; the purpose of the study was to solve water treatment-related problems via the biochemical manufacture and characterization of iron oxide nanoparticles from coated sands.

#### Co-precipitation technique

Wu et al. [110] used the co-precipitation method to synthesize magnetite particles; the co-precipitation strategy used in the study involves varying the reactant concentration and reaction temperature, as these variables affect the process parameters as well as the microstructural and magnetic properties of the base material. The product exhibits high-quality, crystalline NPs with applications in water purification and medication delivery. Chen et al. [111] used extraction solvents to generate iron nanoparticles, and XRD, SEM/EDS, TGA, and FTIR analyses were performed to evaluate the produced magnetite NPs. Tri-butyl phosphate (TBP) and tri-octyl phosphine oxide (TOPO) were utilized as extraction solvents. Both heptanol and 2-ethyl-hexanol are employed as diluents and modifiers [112]. The analysis confirms that after magnetite nanoparticles were exposed to various solvents, the Scherrer equation [113] demonstrated that all investigated extraction solvents had a substantial effect on the sample's mean crystallite size. Co-precipitation production of iron oxide nanoparticles uses hydrated ferric chloride [114], deionized water [115], and ammonium hydroxide [116] as chemical reagents. Magnetite crystals are produced by the ionization of chemical reagents in the presence of nitrogen gas at varied temperatures and with strong stirring [117]. Hence, the benefits of co-precipitation over alternative biochemical approaches for the synthesis of iron oxide nanoparticles include its simplicity, fast preparation at low temperatures, and energy efficiency [118].

#### Reverse co-precipitation technique

Estelrich et al. [119] completed a reverse chemical co-precipitation synthesis using an active reduction reagent as a stabilizer on magnetite (Fe_3_O_4_) NPs. The characteristics of Fe_3_O_4_ and Fe_3_O_4_ NPs coated with dimethyl sulfoxide (DMSO) were compared with those of the uncoated material. Using X-ray diffraction (XRD) and scanning electron microscopy (SEM), the microstructural and mineralogical features of both samples were investigated. The results demonstrated that DMSO-coated sample particles respond better to Fourier studies and SEM examination than uncoated Fe_3_O_4_ nanoparticles [120]. Co-precipitation and reverse co-precipitation procedures are time-consuming, have repeatability issues when employing base materials with varying reactant rates, and have the potential to introduce foreign particles into the metal matrix structure of the iron oxide nanoparticle product [121].

#### Hydrothermal technique

The hydrothermal approach for generating iron oxide nanoparticles by oxidizing iron has gained remarkable popularity among nonscientists in recent years [122]. The hydrothermal synthesis of iron oxide nanoparticles has applications in the fields of superconductors, microporous crystals, complicated iron oxide ceramics for solid electronic conductors, and magnetic materials. The hydrothermal breakdown involves a heat treatment in which iron nanoparticles ranging in size from 15 to 25 nm are generated [123]. Under certain temperatures and pressures, nanoparticles of iron oxide are produced in a reactor through crystallization. The manufactured nanoparticles are crystal growth products derived from warmed iron oxide materials employing organic solvents and precursor solutions as reagents. Hydrothermal processing of iron oxide nanoparticles has the advantage of producing homogeneous nanoparticles with reduced size, shape dispersion, and uniform nanostructure crystallinity [124]. Nanoparticles must be created in high-temperature, high-pressure reactors, which has significant financial ramifications.

#### Sputtering technique

Sputtering is an atomic ejection-based process in which charged particles are ejected off the surface of a substance. It is a phenomenon that occurs when a particle with extremely high kinetic energy strikes the surface of a metallic material [125]. On the substrate layer of the targeted material's surface, sputtered atomic particles are deposited. There are three types of sputtering procedures for atomic particle ejection from their base materials: which include reactive sputtering, DC sputtering, and magnetron sputtering. This includes reactive sputtering, in which an inert gas (such as argon) is employed as the sputtering gas and is activated [126]. At all costs, avoid the reaction between the inert gas and the sputtered atom. In this procedure, powered sputtering gas or inert gas is used to remove oxides and nitrides off the surface of metals (i.e., iron oxide). DC sputtering is yet another form of sputtering technology [127]. This occurs when a substantial number of charged particles expel ionized atoms (or molecules) (i.e., plasma). After the energized ions from the plasma contacted the target, the charged particles returned to their starting state of atomic neutralization. The neutralized ion is deposited as nanoparticles on the surface of the target substance. The shapes and sizes of nanoparticles created by the aforementioned sputtering technique depend on the substrate's temperature, layer thickness, and annealing time. Peng et al. [128] and Roy et al. [129] have examined thin films deposited by an iron oxide target by sputtering. A blend of hematite, magnetite, and a silicon or glass substrate composes the foundation material. They found that the particles exhibit critical thickness growths as a result of a phenomenon known as substrate bias, which enhanced their magnetic properties. Sputtering techniques for creating IONPs are adaptable to nearly all material kinds, but their basic target materials are frequently costly. Sputtering target poisoning must be prevented through the use of a preventative control mechanism. [77]. Using a high-resolution transmission electron microscope (HRTEM), the crystallographic defect observed on the film surface of the produced iron oxide nanoparticles [130] was investigated. Several works of literature [131–133] have confirmed that metallic nanoparticles (NM) created by the sputtering approach have superior magnetic and microstructural properties compared to those produced by other bottom-up procedures. The greatest benefit of nanoparticles created by the sputtering technique is their adaptability to nearly all material kinds [134]. It is possible to manage the material composition to avoid unanticipatedly complex chemical processes. The downside of sputtering approaches for IONPs is that their basic target materials are frequently costly [135]. NPs are not energy-efficient; thus, sputtering target poisoning must be prevented through the use of a preventative control mechanism. Additional biological techniques for the creation of iron oxide nanoparticles include spark discharge [136], micro-emulsion [137], microwave-assisted methods [138], laser ablation [139], ultrasound [140], and the sol–gel approach [141].

### Synthesized iron oxide nanoparticles’ mechanophysical method

Sahoo et al. [142] stated that the necessity of producing iron oxide nanoparticles stems from the fact that oxidizing iron into IONPs modifies its characteristics. According to scientific research, metallic oxide NPs are more reactive than their base metals [143]. This indicates that iron oxides are more reactive than nanoparticles of iron [144]. Thus, IONPs are often created by thermal decomposition, lithography, sputtering, and mechanical/ball-milling. These procedures are utilized as convectional physical procedures to create IONPs.

#### Mechanical/ball-milling technique

Arbain et al. [145] synthesized α-Fe_2_O_3_ from natural iron ore using the mechanical alloying technique. Using SEM, XRD, and XRF, the morphological, mineralogical, and phase composition of the produced Fe_2_O_3_ samples were analyzed. The XRD and XRF analyses of the synthesized sample reveal similar and consistent properties. Hence, the study demonstrates that the nano-hematite (α-Fe_2_O_3_) phase formed by the mechanical milling process plays a crucial role in the magnetic behavior of its natural ore sources [146]. To determine the magnetic properties of nanoparticles, Chen et al. [147] produced magnetite NPs using the ball-milling process. The phase structure and mineralogical characteristics of the NPs were determined by XRD and XRF analysis. NPs with strong magnetic properties are the outcome of the stoichiometric mixing of hematite samples with metallic iron particles in an inert atmosphere over a predetermined period. The study provides additional support for the indisputable influence of magnetic properties and hardness on magnetite nanoparticles produced by ball milling in the presence of a hot, inert gas atmosphere.

#### Heat treatment/thermal decomposition technique

In general, the thermal decomposition heat treatment technique can be used to synthesize nanomaterials from iron oxides at temperatures exceeding 250 °C [148]. The technique of thermal decomposition is an endothermic procedure involving a chemical reaction. The chemical production of nanoparticles occurs when chemical bonds, or van der Waal forces, are disrupted by a hot reaction [149]. This heat of reaction is the temperature at which the chemical elements decompose. Using the thermal breakdown approach on iron oxides invariably results in the presence of NPs, which are further synthesized into nanomaterials according to their uses [150]. The produced nanoparticles are more beneficial in superparamagnetic, biomedical, drug delivery, and wastewater treatment applications of which FeNPs and IONPs are such nanomaterials. The limitations of superparamagnetic iron oxide nanoparticles are their inability to be stored for an extended period, their poor maintenance structures when agglomerated, and their instability [151].

When iron ore is heated to 265 °C, the general breakdown method creates monodispersed iron oxide nanoparticles, which can be functionalized by the direct reduction of single metal ions on the surface of IONPs at a high temperature [152]. At an annealing temperature of 250 °C, magnetite NPs decompose readily into maghemite (γ-Fe_2_O_3_) NPs via an oxidation reaction [153]. Sun et al. [154] and Shao et al. [155] studied the size-controlled mechanism of monodispersed Fe_3_O_4_ NPs by the thermal decomposition technique. The monodispersed character of the nanoparticles is a clear indicator that the magnetite crystal structure is changed into α-Fe_2_O_3_ nanoparticles via thermal decomposition without the need for size reduction [156]. Consequently, functionalized iron oxide nanoparticles can be generated by the direct reduction of single metal ions on the surface of IONPs at a high temperature.

#### Lithographic technique

The lithographic process is one of the physical approaches used to produce nanoparticles. The procedure has proven to be highly costly and energy-intensive over time [157]. Nanomaterials created by the lithographic process have several uses in electronic devices and computer accessories. Lithographic synthesis is a top-down technique that can be utilized to create micro- and NPs. Fusion-ion lithography, electron beam lithography, photolithography, nano-imprint lithography, and dip-print lithography are some of the known diverse methods of synthesis [158]. It has numerous uses, including additive manufacturing and semiconductors.

### Parameters of synthesized IONPs

#### Size and shape of IONPs

Generally, most production parameters involved in iron or ferrous metal-related nanofabrication processes are interdependent. Therefore, it is important to understand the correlation between other nano-processing parameters and the precise size of iron oxide nanoparticles. For instance, the ferromagnetic behavior of maghemite and magnetite is similar when their particles are synthesized at a nano-sized range between 15 nm and − 45 nm [159]. Also, nano-size variations of IONPs are produced based on their functionality and applications in many known conventional processes, especially in magnetic and biomedical-related fields [160]. Therefore, to fully ascertain the precise size of iron oxide nanoparticles, it is important to understand the correlation between other nano-processing parameters (i.e., shape, magnetic property, etc.) and their various areas of applications [161, 162]. For example, at the magnetism saturation point of 92 emu g^−1^, γ-Fe_2_O_3,_ Fe_3_O_4,_ and α-Fe_2_O_3_ revealed that both nanoparticles do exhibit weak ferrimagnetic and ferromagnetic tendencies at room temperature, respectively [163]. Furthermore, the superparamagnetic properties of iron oxide nanoparticles are revealed whenever their synthesized size is below 15 nm [11]. Also, depending on the degree of energy barrier due to anisotropic properties over thermal energy, IONPs can be processed to have NPs of 13–18 nm with better magnetic NPs in a size range of 13–18 nm and demagnetization tendencies [164]. Xing et al*.* [165] and Vergnat et al*.* [166] studied the production of iron nanoparticles from Fe_3_O_4_ particles using a high-pressure gas condenser surrounded by a maghemite shell. The study revealed that the synthesized iron core nanoparticle has a size range of 10–20 nm. The shape and size of the iron nanoparticles are strongly influenced by the energy generated by the gas environment. The overall particle behavioral characteristics of the produced nanoparticles were investigated based on their superparamagnetic fractions and magnetic susceptibility to frequency dependence.

Lesiak et al*.* [167] studied the systematic production of FeNPs from recently discovered Fe_3_O_4_ particles of distinct size ranges 85–5 μm in the Coloradan iron ore deposit in Mexico. The changes in mineralogical and morphological characteristics of the magnetite particles were done using XRD and Mossbauer spectroscopy. High-resolution transition electron microscopy (TEM) was used to investigate the crystallographic changes in sizes of the magnetite particles upon transition from micro-metric to nanometric structures [168]. The overall particle behavioral characteristics of the produced nanoparticles were investigated based on their superparamagnetic fractions and magnetic susceptibility to frequency dependence. In addition, the isothermal efficiency, coactivity, and remnant magnetization properties of the IONPs were investigated [128, 169]. The study of NP sizes as reported in the literature reveals that the direct magnetic separation method could serve as an alternative approach to the production of metallic iron NPs, especially for Fe_3_O_4_ materials that are acquired as run-off mines [170].

Another approach to ascertain the size and size distribution of IONPs is the use of morphological analysis of the synthesized particles using a transmission electron microscope (TEM), scanning electron microscope (OPM), and optical imaging micrograph (OPM). IONP size distribution within the metal matrix revealed the more precise size and shape of the grain boundary by intergranular paths along with the grain size [171, 172]. The overall nanoparticle behavioral characteristics of the produced nanoparticles were investigated based on their superparamagnetic fractions and magnetic susceptibility to frequency dependence [138, 173], and recent advances have been made in the areas of nano-imaging [174], magnetic resonance imaging [175], and size control [176] with diverse applications found mainly in biomedicine and drug delivery systems. In addition, IONP size separation [177] using TEM, where nanoparticles of similar shapes with almost the same size range are coerced or condensed within the functionary surface of the IONP metal matrix, can be classified based on their grain sizes and particle density within the phase metal matrix structure of the Fe-alloyed using the composite materials magnetic resonance imaging (MRI) technique [178].

#### Magnetic properties of IONPs

Numerous published articles have proposed a scientific strategy for transforming conventional IONPs into superparamagnetic IONPs via surface functionalization techniques. Loss of magnetism and disparity have been regarded as insurmountable obstacles for magnetic IONPs. These strategies include shell–core–shell, matrix dispersion structure, core–shell structure, and Janus-type structures, among others. The Janus structure improves the magnetic behavior of IONPs' base materials under the influence of a magnetic force field by employing functionalized magnetic IONPs as stabilizing nanomaterials. Loss of dissimilarity in magnetic nanoparticles is the ability of magnetic NPs to have less surface energy within their magnetic force field when smaller NPs join together to make larger particles for magnetic resonance imaging [179]. Also, the loss of magnetism is accompanied by chemical activity inside the metal matrix of IONPs. This activity destabilizes the NPs' crystal structure, which makes them less useful [180]. Several publications [181] have suggested different techniques to fix the structural damage that IONPs obtain from chemical activity, very large energy, and particle aggregation to fix the problem with their magnetic behavior. Hence, the damage restoration approach includes shell–core–shell, matrix dispersion structure, core–shell structure, and Janus-type structures, among others [182].

The core–shell structure involves encapsulating a microstructure of iron oxide with nano-coating materials [183]. These coating materials exist in the form of magnetic composites that travel into the nuclei of the IONP to fill the space within the crystal structure of the IONP's base materials and create stability within its elemental core shells. In the shell–core–shell structural strategy, two functional materials are positioned next to one another on magnetic IONPs [184]. These materials provide crystal support for the magnetic IONPs by shielding their core from chemical interactions that could compromise their colloidal stability inside their metal matrix crystal structure. These surface functionalization and protection materials, sometimes known as "shells," prevent any undesirable interactions or chemical activity from penetrating the core of IONPs. In addition, the physical strategy for performing the matrix dispersion experiment necessitates preventing tiny superparamagnetic nanoparticles from growing into larger but weaker magnetic IONPs [185]. In contrast, the Janus structure solves the issue of loss of disparity and magnetism in IONPs by employing functionalized magnetic IONPs as stabilizing nanomaterials, which improve the magnetic behavior of the IONPs' base materials under the influence of a magnetic force field. The Janus particle's magnetic field eliminates discrepancies and enhances the magnetic characteristics of the IONP's base material. The Janus structure modifies the microstructure of the IONP's base materials [186]. Thus, the microstructure of the IONPs' base material must be investigated to comprehend various methods for enhancing the magnetic properties of IONPs and resolving issues related to their superparamagnetic capabilities, such as loss of disparity and loss of magnetism.

#### Microstructure and morphological properties of IONPs

**Fig. 5:**
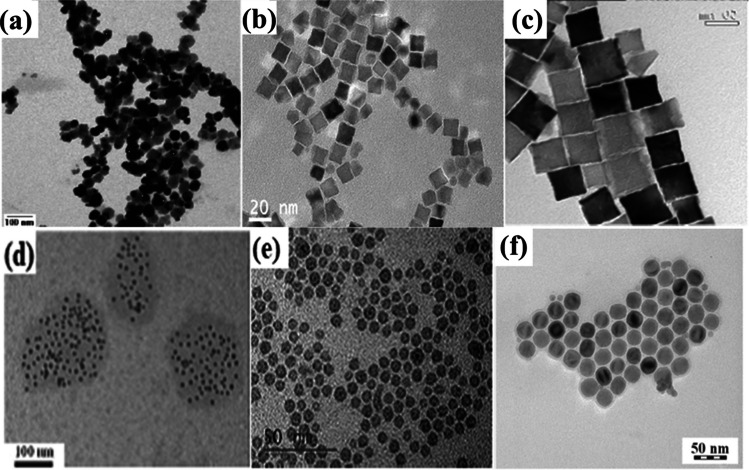
TEM micrographs of IONPs (Fe_3_O_4_/*γ*-Fe_2_O_3_) with size range **a** 5–26 nm hydrothermal [23], **b** 5–24 nm thermal decomposition [32], **c** 4–20 nm, thermal decomposition [33], **d** 13–25 nm, physiochemical method [34], **e** 3–11 nm rapid induction heating method [35], **f** 10–24 nm, co-precipitation technique [40]. Copyright: 2009, 2011, 2020 American Chemical Society; 2012, 2015, 2018 Elsevier

**Fig. 6:**
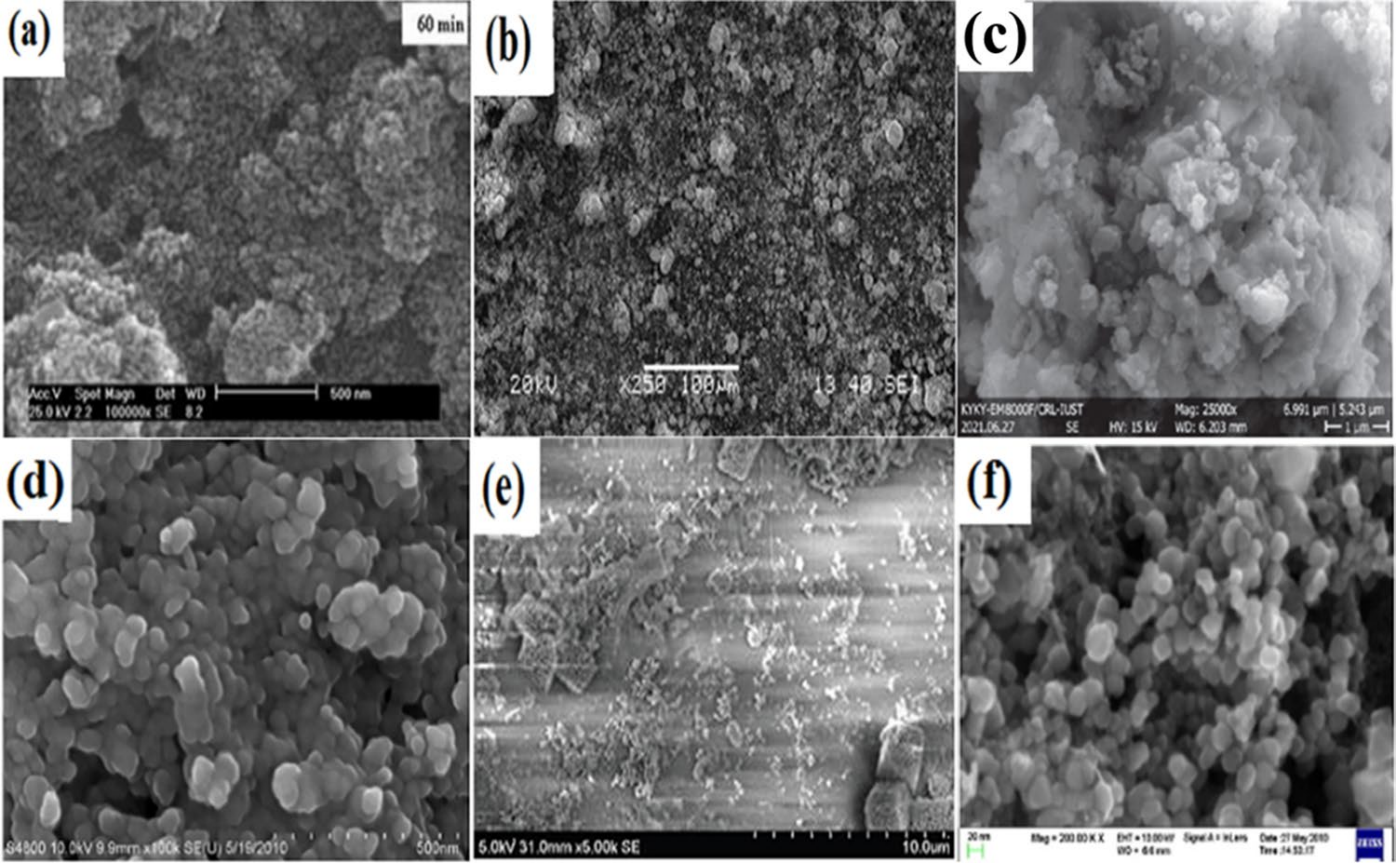
SEM micrographs of IONPs at 60 min **a** *α*-Fe_2_O_3_, mechanochemical process [147] **b** α-Fe_2_O_3,_ mechanical milling [151] **c** CoFe_2_O_4_, mechanical milling [156] **d** Fe_3_O_4_, co-precipitation [149] **e** Fe_3_O_4_, sol–gel method [158] **f** Fe_3_O_4_, chemical co-precipitation [159]. Copyright: 2008 American Chemical Society; 2009, 2011 Springer Science; 2012 Trans Tech; 2016, 2021 Elsevier

Most FeNPs and IONPs are characterized using SEM/EDS, XRD, TEM, etc., which are all powerful electromagnetic devices. Experiments are carried out under very specific conditions. Sun et al. [187] produced nanoparticles of magnetite iron oxide through the hydrothermal technique and examined their morphology in terms of the atomic or crystalline grain arrangements of the produced nanomaterials. The SEM image of the synthesized IONPs demonstrated the production of a highly crystalline particle with a considerable change in particle size (from 15 to 31 nm). Figures 5 and 6 depict the TEM and SEM micrographs and morphology of several IONPs manufactured in various published publications using various nanofabrication methods [188]. IONPs are typically subjected to TEM analysis to examine their morphology in terms of the atomic or crystalline grain arrangements of the produced nanomaterials [189]. Using the principle of electron diffraction of NPs, the morphology of selected portions of the phase microstructure is illustrated. Figure 5(a) depicts a TEM picture of synthesized magnetite exhibiting an ellipsoidal particle form. IONPs produced by the hydrothermal technique have a typical Fe_3_O_4_ nanostructure, while IONPs generated under high-temperature conditions have a dispersed microstructure with smaller particle sizes. The size and shape of manufactured IONPs are loaded into nanocarriers without altering their mechanochemical properties. In contrast, IONPs synthesized using the thermal breakdown approach, as represented in Fig. 5(b), have a dispersed microstructure with smaller particle sizes, indicating that IONPs generated under high-temperature conditions contain nanoparticles with smaller sizes. At room temperature, the size dependency of high-quality α-Fe_2_O_3_ NPs is examined in Fig. 5(c) using the thermal decomposition approach. Notable is the fact that adjusting the ambient temperature of the manufactured IONPs may affect the magnetic and sizing properties of the nanoparticles. The IONPs underwent a change that modified the crystallinity structure from α-Fe_2_O_3_ to Fe_3_O_4_. The TEM picture of IONPs was created using the physicochemical technique, as depicted in Fig. 5(d). The micrograph also displays the number of IONPs contained within the nanocarrier. The size and shape of manufactured IONPs are loaded into nanocarriers without altering their mechanochemical properties. This implies that IONPs generated by inductive heating are monodispersed and uniformly distributed [190]. Figure 5(e) depicts the characterization of the TEM image of IONPs generated by inductive heating. In the crystals of the microstructure, the synthesized IONPs are monodispersed, uniformly distributed, and have averagely lower dimensions than IONPs generated by other known conventional techniques. IONPs generated by chemical co-precipitation have a consistent size and comparable magnetic and chemical properties. Depending on the reaction precursor and time, the synthesized NPs produced by these procedures have averagely lower dimensions. In comparison with other known conventional techniques of IONP synthesis, IONPs synthesized by the inductive method have been demonstrated to be safer and easier to duplicate at an industrial scale. Accordingly, Fig. 5 depicts the size dependence of the IONPs generated by chemical co-precipitation Fig. 5(f). The nanostructure of IONPs produced by the co-precipitation process demonstrates their monodispersed crystal structure [191]. Hence, IONP generated using this method has a consistent size and comparable magnetic and chemical properties. Similarly, TEM examination of certain IONPs produced using well-known conventional techniques revealed that the nanocrystals were homogeneous and possessed consistent properties. These characteristics are highly unique to the vast majority of IONP types, their synthesis, and their characterization methodologies, regardless of the iron oxide base materials employed.

In addition, IONPs (Fe_3_O_4_/Fe_2_O_3_) can be synthesized by hydrothermal, thermal decomposition, physiochemical, and rapid induction heating methods. Anjum et al. [192] synthesized IONPs using the solid-state chemical reaction movement technique, SEM/EDS, and thermogravimetric analysis (TGA) at a temperature range of 50–800 °C. The milling time for synthesized IONPs ranges between 30 and 60 min. The SEM micrograph illustrated in Fig. 6(a) demonstrates that the phase of IONPs remains stable as particle size decreases with time. The SEM results concur with the XRD findings. When the TGA temperature rises, the iron phase of the produced nanoparticles changes. Hence, α-phase IONPs transform into γ-phases IONPs as the reaction temperature transitions from endothermic to exothermic and reaches 800 °C within 6 min. In Fig. 6(b), the Fe_2_O_3_ particle size was produced by mechanical milling for 15–60 min. The iron oxide phase of the described nanoparticle displays bulky or compacted grains with a strong oxygen trace [193]. The SEM result shown in Fig. 6(b) is consistent with the results reported in the following figures, which demonstrated that the phase IONPs remain unchanged when the particle size decreases with milling time. This suggests that the dense microstructure phase of CoFe_2_O_4_ produced by mechanical milling, as depicted in Fig. 6(c), validates the nature of cobalt (Co) when burned in the presence of oxygen. Due to the presence of cobalt inside the produced IONP metal matrix, the iron phases of Fig. 6(c) display an ice-cake-like appearance with a dense, whitish microstructure. Figure 6(d) is the SEM micrograph of IONPs that were chemically produced utilizing the co-precipitation method. The properties of the presented SEM micrograph reveal a grain resembling a dense cake, as the IONPs phase is a dense structure devoid of microspores. Figure 6(e) shows the SEM image of the synthesized IONPs, which are chemically produced by the sol–gel biochemical technique, and coated with sand. Due to the presence of silica particles in the coated sand, the morphology displays a spherical iron-phase microstructure surrounded by monodispersed micropores, which promotes stability and shields the NPs from acidic conditions. In contrast, Fig. 6(f) displays a fibrous crystal structure of the iron oxide phase with widely spread, uniform-sized micropores. The properties of the presented SEM micrographs reveal a grain resembling a dense cake devoid of microspores, and a fibrous crystal structure of the iron oxide phase with widely spread, uniform-sized micropores. The scattered character of the Fe_3_O_4_ phase may have been the outcome of the coated chemical agent's chemical action (i.e., C_12_H_25_OSO_3_Na). Hence, the SEM micrographs of Figs. 6(a) through 6(f) reaffirmed the importance of the synthesis method, coating materials, temperature variations, and chemical agents utilized in the nanofabrication and nano-processing of metals and metallic alloys. Depending on the physical, chemical, and biological properties of the starting material, these processes have enormous effects on the nanoparticles' size, shape, magnetic behavior, morphology, and microstructure.

#### Biocompatibility effect and biological activity of IONPs

**Fig. 7:**
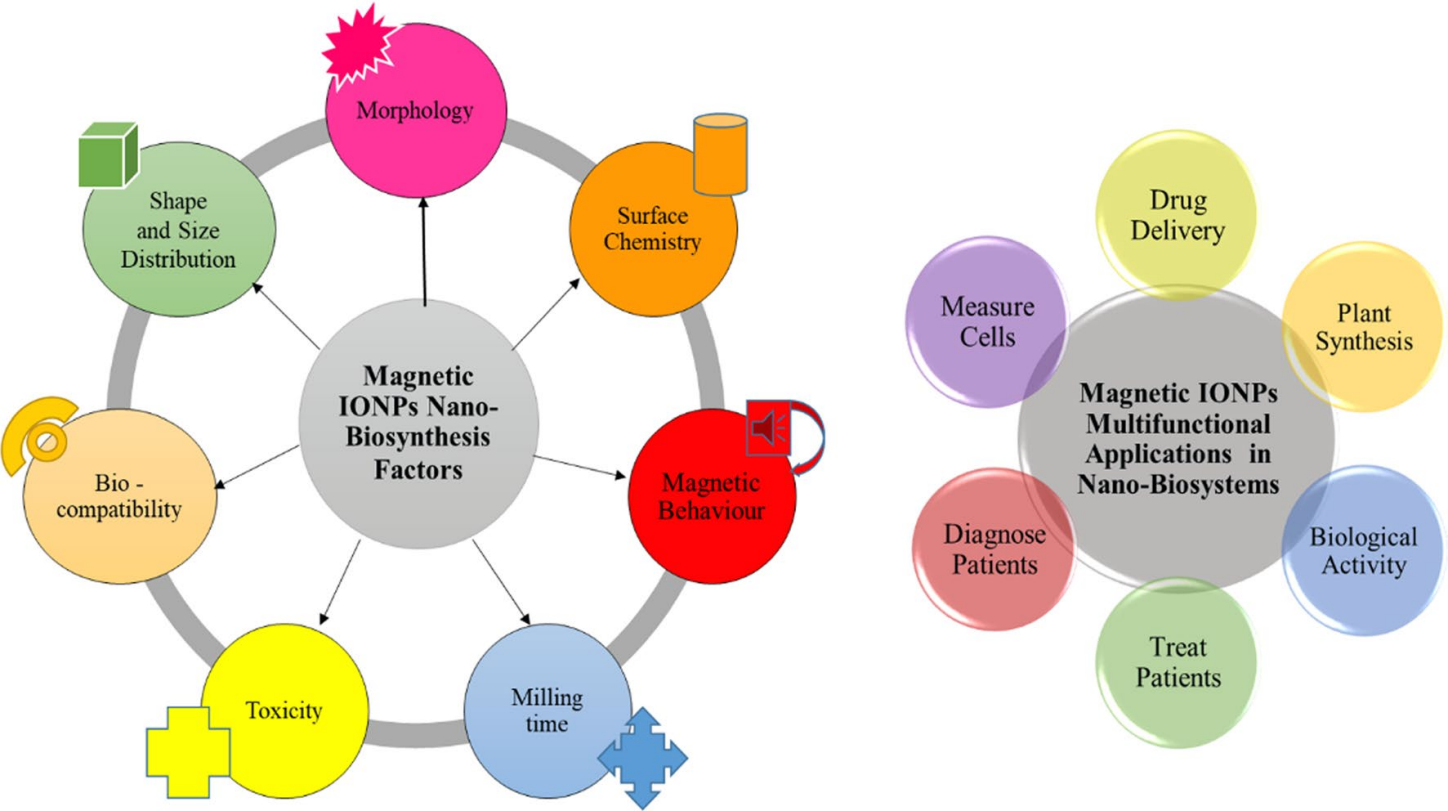
Schematics of magnetic IONPs synthesis, influential factors and applications in nano-biosystems

Magnetic iron oxide nanoparticles (MIONPs) have proven to be quite suitable for biological and biomedical applications because of their good saturation magnetization and high magnetization moment. These MIONPs include maghemite and magnetite. The importance of magnetic IONPs is mostly attributed to so many factors, which are in turn based on the method of synthesis and the proposed areas of application. These factors include size distribution, morphology, surface charge, surface chemistry, capping agents, etc. (see Fig. 7). MIONPs have also been exploited as model organisms in biosystem multifunctional nanomaterials based on their biological activity, which includes antibacterials, toxicity, and drug delivery [194–196]. The biological activity and biocompatibility of MIONPs can be influenced by their morphological properties, particle size, and magnetic behavior [197, 198]. This often occurs during synthesis based on the nanocrystalline phase transformation characteristics and growth mechanism. The changes in properties of MIONPs based on their nanocrystals' growth mechanism and phase transformation mostly occur through the nucleation process during synthesis [199].

**Fig. 8:**
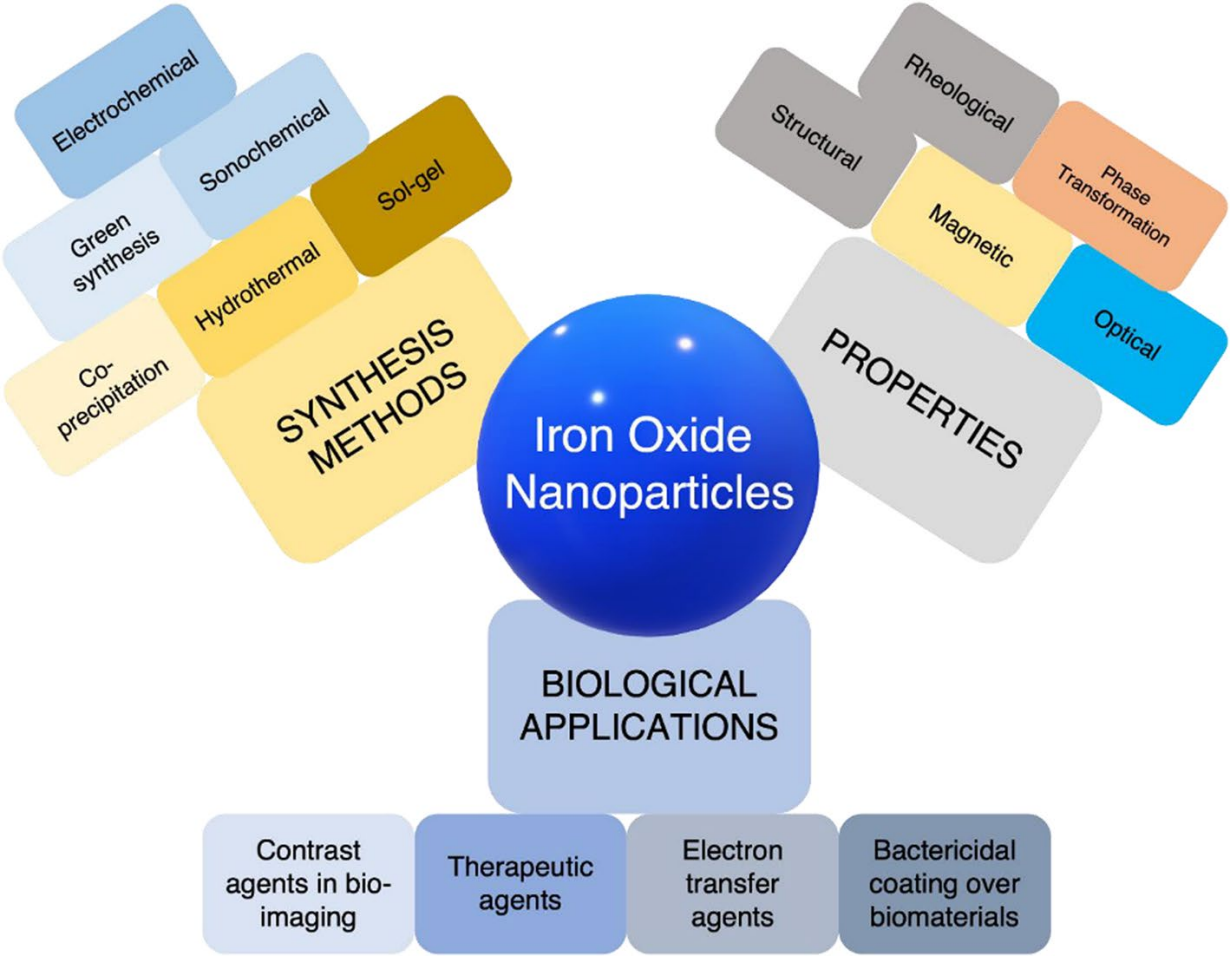
Schematics of synthesis of IONPs using biological method and application [203]

For instance, the mechanism of action during biological activities in MNIOPs entails a nano-interaction process between the crystal lattices in living cells and energy changes by the nucleation process. This involves the promotion of catalytic activities in enzymes to enhance or cause performance reduction in the cells [200]. Also, this interaction could lead to a malfunction or death of such living cells because of the energy changes caused by cell disruption integrity, which may occur within the cell membrane [201]. The synthesis of IONPs using biological methods is often referred to as green synthesis. It has several advantages over the traditional physicochemical methods. Figure 8 depicts the overview of the superiority of the biosynthesis method based on green nano-processing and application. The superiority of biologically synthesized IONPs over the physicochemical method is based on their wide applicability, cost-effectiveness, biocompatibility, reduced toxicity, narrow size distribution, environmentally friendly, reduced energy consumption, scalability, bio-functionalization, and biological catalysis tendencies [202].

The eco-friendly factor of the biosynthesis method of IONPs utilizes natural microorganisms and compounds to minimize hazardous waste and environmental impact and reduce the need for harsh chemicals and high temperatures in nanoparticle synthesis [204]. In the biological synthesis of IONPs, there is less likelihood of adverse reactions in living organisms because the use of biocompatible materials results in IONPs suitable for biomedical applications, imaging, and drug delivery systems with lower toxicity [205]. Also, biosynthesized IONPs have a biocompatibility advantage with enhanced surface modifications ensuring their suitable applications in pharmaceutics and biomedicine [206]. IONPs are mostly synthesized by mechanochemical process, mechanical milling, co-precipitation, sol–gel method, and chemical co-precipitation. However, these methods can promote or cause performance reduction in the cells, even malfunction or death of such living cells. IONPs synthesized by biological methods can yield narrow size distribution properties, which are essential for application in MRI contrast agents with uniform-sized NPs for better imaging quality [207]. To allow IONPs to suit some specific applications, the biological synthesis method can be fine-tuned to control the size, shape, and excellent multifunctional properties. Also, the use of microorganisms in the biosynthesis of IONPs can often be cultivated inexpensively. Thus, biological synthesis is less expensive than physicochemical methods because they require fewer chemicals and energy-intensive processes. Invariably, green synthesized IONPs biological synthesis at lower temperatures and pressures reduces energy consumption compared to high-temperature physicochemical methods. This lesser economic implication can be adapted for large-scale production as a result of reduced energy consumption, lower temperature, and pressure required during production, which is most crucial during industrial applications [208]. The bio-functionalization factor associated with IONPs is synthesized biologically for application in biomolecules (i.e., antibodies and enzymes) without any need for additional chemical modifications. In addition, the catalytic properties of biologically synthesized IONPs using microorganisms and enzymes can be highly efficient in reducing the need for chemical reagents and promoting the formation of such NPs. Thus, the superiority of biologically synthesized IONPs over physicochemical methods is dependent on specific requirements and applications in the modern-day nanofabrication process. The biological activities of IONPs can be maintained by molecule traffic control and cell protection mechanisms within and outside the cell membrane. These biological activities are better explained during the application of such IONPs in drug delivery systems, cancer cell eradication, and other biomedical-related processes as discussed in detail in the upcoming section.

## Applications of synthesized iron oxide nanoparticles

Nanomaterials are created on purpose by chemical reactions or physical actions using engineering techniques, and they exhibit unique physical and chemical properties. They are used in engineering, biology, pharmacology, and environmental remediation. Table 1 contains examples of IONPs and composite-based IONPs used for various engineering and medicinal applications. Regardless of the numerous uses of synthesized IONPs, such as plant and tissue remediation, biomedical treatments, pharmaceutics and drug delivery systems, and wastewater treatment, it is essential to recognize that the need for nanomaterials cannot be overstated. Thus, the part that follows describes in detail the endeavors and achievements of researchers regarding the applicability of IONPs in engineering, biology, pharmacology, and environmental remediation procedures.

**Table 1:** Different IONPs synthesized under physical, chemical, and biological methods and their applications

Nanomaterials	Synthesis methods	Applications	References
Fe_3_O_4_	Chemical co-precipitation	Composite materials	Gopal et al. [1]
Fe_3_O_4_	Mechanical and microstructure characterization	Multifunctional materials	Rivas-Sanchez et al. [2]
γ-Fe_2_O_3_	Crushing, sieving, solvent extraction, and co-precipitation	Water purification and environmental remediation	Sebehanie et al. [3]
Fe_3_O_4_; α-Fe_2_O_3_	Thermal decomposition and magnetic resonance	Biomedicine and cancer treatment	Wierzbinski et al. [4]
Fe_3_O_4_	Hydrothermal synthesis	Multifunctional materials, biomedical applications, and Cancer treatment	Li et al. [5]
α-Fe_2_O_3_	Reversible Aggregation and Magnetic Coupling	Multifunctional materials and electronic appliances	Frandsen et al. [8]
Fe_3_O_4_-Silica	Reverse-micelle technique	Biocatalysis and bioseparation applications	Yang et al. [10]
Fe_3_O_4_; nano-powder	Ultrasonic-assisted chemical co-precipitation method	Magnetic materials and iron ore tailing applications	Wu et al. [11]
Fe_3_O_4_; fine particles	Sol–gel reaction method	Multifunctional materials and data storage devices	Lee et al. [12]
γ-Fe_2_O_3_	Inductive Heating Method	Chemical synthesis and monomer concentrations	Lian et al. [13]
Fe_3_O_4_@AuNPs	Sonochemical method	Breast cancer treatment	Dheyab et al. [48]
Fe_3_O_4_NPs	Simple rapid stabilization method	Breast cancer treatment	Dheyab et al. [48]
Fe_3_O_4_@AuNPs	Sonochemical method	Magnetic resonance imaging (MRI) and CT scan	Dheyab et al. [49]
Fe_3_O_4_@AuCS	Rapid sonochemical synthesis	Cancer cell eradication and biomedical applications	Dheyab et al. [50]
Fe_3_O_4_NPs, AuNPs and Fe_3_O_4_@AuNPs	Sonochemical method and Response Surface methodology	Drug delivery, MRI, and Biomedical applications	Dheyab et al. [53]
Fe_3_O_4_	Biochemical synthesis	Drug delivery systems	Maharramov, et al. [64]
Fe_2_O_3_; Suspension	Humic acid leaching and absorption process	Subsurface water and soil treatment	Ahmed et al. [65]
FeO_x_ film	Sputtering and Plasma emission monitoring	Magnetic storage, electric devices, and iron ore tailing applications	Aubry et al. [84]
α-Fe_2_O_3_ and γ-Fe_2_O_3_	Laser ablation	Hyperthermia application	Rivera-Chaverra et al. [88]
Fe_3_O_4_	Wet mechanical milling process	Wastewater purification and environmental remediation	Chen et al. [97]
Fe_3_O_4_	thermal decomposition	Magnetic storage, electric devices, and iron ore tailing applications	Maity et al. [100]
Fe(CO_3_)	thermal decomposition	X-ray scattering, Magnetic data storage Magnetic Resonance Imaging (MRI)	Lassenberger et al. [105]
Fe_3_O_4_; Magnetic	Ultrasonic-assisted chemical co-precipitation	Magnetic materials	Wu et al. [110]
Fe_3_O_4_; Magnetic Hydrophobic	Biosynthesis	Tumor malignant detention and Vivo-Magnetic Resonance Imaging (MRI) process	Chen, et al. [111]
Fe_3_O_4_; fine particles	Co-precipitation method	Magnetic storage devices	Li et al. [114]
α-Fe_2_O_3_	Mechanochemical Synthesis	Multifunctional materials	Seyedi et al. [157]
γ-Fe_2_O_3_	Sol–gel reaction method	Magnetic storage devices	Kayani et al. [158]
Fe_2_O_3_ and Fe_3_O_4_ SPION	Chemical co-precipitation	Biomedical and Magnetic Resonance Imaging (MRI) and Magnetic Particle Imaging (MPI)	Dadfar, et al. [168]
Fe_3_O_4_; Ferrofluid	Chemical co-precipitation	Micro actuation process	Pislaru-Danescu et al. [170]
γ-Fe_2_O_3_	Chemical co-precipitation	Removal of heavy metals and Industrial wastewater	Cheng et al. [138]
FeCl_3_ solution	Biosynthesis	Antibiotic production or microbial research	Honary et al. [243]
Fe_2_O_3_	Biosynthesis	Antimicrobial agents and Drug delivery systems	Kahn et al. [244]
AuFe_3_O_4_ AgFe_3_O_4_	Biosynthesis	Antimicrobial testing, Prostate-specific and protein antigen detection	Jung et al. [245]
Fe_3_O_4_	Biosynthesis	Photocatalytic degradation, Antibacterial agent, and Wastewater filtration	Mahlaule-Glory et al. [246]

### Remedy for bone repair and tissue degradation

IONPs are useful in tissue engineering and cell regeneration, as well as in horticulture and cancer patient treatment for plants and humans, respectively. However, additional research on IONPs' duration, size precision, and magnetic field intensity is necessary before their application in gene protein acceleration for bone tissue regeneration and skill remediation. IONPs' repair capabilities and application in horticulture and cancer patient treatment for plants and humans, respectively, are among their numerous advantageous applications [209]. Fathi-Achachelouei et al. [210] provided an overview of iron oxide nanoparticles' applications in tissue engineering and regenerative medicine. Recent breakthroughs in the application of IONPs in convection-based medicinal practices have centered on the use of nanomaterials to monitor skin-related harm in therapeutic medicine [211], cancer therapy [212], and cell regeneration [213]. Yet, the translation of synthesized IONPs into clinical medicine applications continues to be plagued by numerous obstacles. This issue mostly includes toxicity investigations on IONPs, which are crucial for clinical translation [214]. In practice, multi-parameter labeling and inadequate handling of nanomaterials may hinder their clinical transferability for tissue engineering and cell regeneration [215]. Consequently, the successful translation of clinical medicine must involve an in vivo and in vitro toxicological process before the bio-distribution of IONPs in the injured tissue. Bone tissue restoration is another biomedical application of IONPs, as IONPs, with their non-toxicity and good magnetic and semi-conducting properties, are extraordinarily useful for tissue repair [216]. The IONPs are integrated into stem cells, which strengthen the muscle fibers surrounding the bones of affected individuals [217]. Using magnetic action, this combination increases the materialization process of the protein fiber surrounding the afflicted bone area [218]. Before its application in gene protein acceleration for bone tissue regeneration and skill remediation, however, additional research on the IONP's duration, size precision, and magnetic field intensity is necessary.

### Drug delivery systems and cancer treatment

Superparamagnetic IONPs are employed in medicine delivery systems via magnetic resonance analysis and are also used in radiology and pharmaceutical medicine via MRI to treat cancer. Numerous publications have examined the biocompatibility applications of superparamagnetic IONPs and their use in clinical and diagnostic medicine [219]. The use of synthetic magnetic IONPs in drug delivery systems relies on the magnetic resonance analysis concept, in which the action of molecular medicine transports the nanoparticles to the precise location of damaged tissues. Thus, the migration of medications into the afflicted tissue does not necessarily impact the magnetic characteristics of the IONP. In addition, multifunctional IONPs can be used in imaging and drug delivery systems for prostate cancer, and they also have applications in breast cancer medicine delivery systems. Gutierrez et al. [220] investigate the use of such IONPs in MRI and drug delivery systems for prostate cancer. The process is referred to as the double-receptor targeting method, which suggests an alternative to chemotherapy for prostate cancer. By targeting nanoparticle therapy at the damaged region, harmful chemicals delivered by anticancer drugs into cells and tissues are decreased. This approach also has the disadvantages of being expensive, time-consuming, difficult to use, and requiring multiple clinical investigations on the IONPs before usage. Before using IONPs in any form for drug delivery or medicinal applications, the magnetic characteristics must be understood. Failing to determine the magnetic sensitivity of IONPs can result in clinical failure. The most significant benefit of the magnetically sensitive nature of IONPs over convectional physical methods is their versatility of application in drug delivery domains due to their simplicity, low cost, and shortened incubation time [221]. Magnetic IONPs also have applications in breast cancer medicine delivery systems [222]. In this method, pure maghemite (γ-Fe_2_O_3_) is substituted with magnetite NPs, which may produce harmful radicals that degrade into health concerns [223]. Consequently, the growing interest in magnetic nanoparticles (γ-Fe_2_O_3_) as an alternative to magnetite IONP is minimizing the risk of generating hazardous Fe (II) ion radicals on the surface of malignant breast tissue. However, a comprehensive biocompatibility process must be executed on the various types of IONPs for drug delivery and cancer treatment [224] for applications in biomedicine.

### Waste water and environmental remediation

The importance of nanomaterial synthesis, characterization, and application research cannot be overstated. The applications of IONPs are most prominent in wastewater treatment, environmental remediation, pollution prevention, and the sensing and detention of foreign bodies in water bodies purification, environmental remediation, pollution prevention, and the sensing and detention of foreign bodies in the waterways [225]. In terms of nanotechnology, water purification involves the removal of pollutants and the treatment of wastewater [226]. Hence, despite the advancements made in nanotechnology for environmental science and wastewater purification, there is a need for a scientific study aimed at the discovery of high-precision nanomaterials and nanotechnological processes [227]. The nanofiltration technique is the principal technological method for wastewater treatment [228], and it uses membrane filtration to remove numerous pollutants. Magnetic IONPs are used in wastewater treatment to neutralize extremely alkaline groundwater. This approach offers a high level of water purification by removing numerous pollutants [227]. Also, the approach is effective for treating solid waste [229], pathogens [230], monovalent and divalent compounds [231], and wastewater [232].

Powell, et al. [233] utilized IONPs for the treatment of treated wastewater in underflow situations via a magnetic nanoparticle device (MagNERD). The method employs superparamagnetic magnetite that was created, collected, and isolated from improved MagNERD.

Magnetic γ-Fe_2_O_3_ IONPs are utilized by Shipley et al. [70] for the remediation of subsurface soil and groundwater. The low PH value (3–5) of IONPs was utilized to neutralize extremely alkaline groundwater. This method is the standard wastewater treatment and soil remediation method that has been optimized [234–236]. Nonetheless, significant technological improvements have been documented in the exploitation, implementation, and application of IONPs in wastewater treatment [237–239]. Due to their strong reactivity, tiny sizes, and outstanding surface functionality, IONPs perform very well as absorbents in wastewater treatment, according to the vast majority of nanomaterials research [240, 241]. This development in the use of IONPs for waste remediation is based on their participation in magnetic particle generation, flocculation, and ionic and bio-separation processes [242, 243]. The major limitations of IONP application for wastewater and environmental remediation are largely attributable to poor regeneration and reuse of the synthesized IONPs, lack of engagement of IONPs in green technology research, and increased treatment water filtration, modification of biosynthesized materials extraction due to their non-biodegradable nature, and increased treatment water filtration due to the highly insoluble nature of the ionized iron hydroxide (FeOOH) nanoparticle [244–246]. Thus, the development of IONPs for water purification, environmental remediation, and biomedical applications remains crucial for the growth of applications of metallic nanofabricated materials for human health and future nanotechnology trends and advances.

## Conclusion and future perspectives

This work examined how IONPs are manufactured and how they might be utilized in biomedicine, wastewater treatment, and environmental cleanup, among other domains. The paper provided an up-to-date examination of the synthesis, characterization, and application methodologies for iron oxide nanoparticles, as well as new developments in nanofabrication and applications. The literature review supports the numerous iron oxide categories, such as maghemite, hematite, and magnetite, and their preparation processes (i.e., mechanical milling, lithography, thermal decomposition, mechanochemical, co-precipitation method, etc.). This is the conclusion of the study:

- Important factors for the synthesis, characterization, and application of iron oxide nanoparticles include, but are not limited to, nanoparticle sizes, shapes, magnetic properties, and surface functioning. Because the aforementioned parameters are the consequential catalytic activity, microstructure phase, and crystallinity of IONPs, nanotechnology has been demonstrated to be useful and necessary in areas such as wastewater treatment, the creation of superparamagnetic devices, drug delivery, and biomedical applications.
- With the development of sophisticated research and methods in the field of green technology for the manufacture of nanomaterials and nanocomposites, the difficulties and hazards connected with nano-processing iron oxides can be reduced.
- The study explains the dependability of IONPs as regards human and environmental concerns cannot be overstated. Using nanofabrication processes to produce innovative engineering materials can be energy-, time-consuming, and capital-intensive.
- The superparamagnetic properties of IONPs (magnetite and maghemite) have encouraged their suitability in diverse areas of applications, including plant development, biomedicine, drug delivery, cancer cell eradication, and environmental pollutants. These interesting magnetization properties are enshrined in biocompatibility, being low toxicity, environmentally friendly, less time-consuming, and having high biomedicine and pharmaceutical tendencies.
- The promising future of treatment of diseases, microbial pathogens, and cancer cell eradication through the introduction of green synthesized IONPs for drug delivery purposes remains a key aspect of the application of the nanofabrication process. Also, IONPs can be used as an alternative remedy for persistent dye removal from soils and the eradication of environmental pollutants.
- It is important to note that the choice between biological and physicochemical approaches for IONP synthesis is determined by the application's specific requirements. While biological approaches have numerous advantages, physicochemical methods may be preferred in some situations, such as when precise control over nanoparticle properties or specific surface coatings is required. Cost, scalability, and the intended usage of the nanoparticles should all be considered.
- For future studies, the synthesis of iron oxide nanoparticles using environmentally friendly technology has numerous advantages, but it is important to conduct a comparative investigation of IONPs created via green fabrication. In terms of their reactivity, particle stability, phase microstructure, and bio-toxicology features, it is necessary to conduct a comparative investigation of iron oxide nanoparticles created via green fabrication.
- The methods of synthesis, characterization, and applications of IONPs and IONP-based composites, which include AuNPs, MIONPs, Fe_3_O_4_@AuNP, nZVI, and SFMIONPs, were discussed in this paper.
- In addition, the risk evaluation of particle aggregation, material dissolution, and kinetics of synthetic iron oxide nanoparticles must be used to provide a solid foundation for functionalized applications of synthesized IONPs made using the green engineering method.

## Data Availability

Raw data is available upon request from the corresponding author.

## Abbreviations

AuNP: Gold nanoparticles
EDS: Energy-dispersive spectroscopy
DMSO: Dimethyl sulfoxide
HRTEM: High-resolution transmission electron microscope
FTIR: Fourier transform infrared
MRI: Magnetic resonance Imaging
NPs: Nanoparticles
Fe_3_O_4_@AuNP: Iron oxide-coated gold nanoparticles
IONPs: Iron oxide nanoparticles
Fe_3_O_4_NP: Magnetite nanoparticles
MIONPs: Magnetic iron oxide nanoparticles
nZVI: Nanoscale zero-valent iron
SEM: Scanning electron microscope
SFMION: Surface-functionalized magnetic iron oxide nanoparticles
SPIONs: Superparamagnetic iron oxide nanoparticles
TEM: Transmission electron microscope
TBP: Tri-butyl phosphate
TGA: Thermogravimetric analysis
TG–DTA: Thermogravimetric/thermal differential analysis
TOPO: Tri-octyl phosphine oxide
XAS: X-ray absorption spectroscopy technique
XRD: X-ray diffraction
XRF: X-ray fluorescence
ZVMI: Zero-valent metallic iron

## Code availability

Source code is available upon request from the corresponding author.
